# Gut Colonization with Methanogenic Archaea Lowers Plasma Trimethylamine N-oxide Concentrations in *Apolipoprotein e*−/− Mice

**DOI:** 10.1038/s41598-018-33018-5

**Published:** 2018-10-03

**Authors:** Ali Ramezani, Thomas D. Nolin, Ian R. Barrows, Myrna G. Serrano, Gregory A. Buck, Renu Regunathan-Shenk, Raymond E. West, Patricia S. Latham, Richard Amdur, Dominic S. Raj

**Affiliations:** 1 Division of Renal Diseases and Hypertension, The George Washington University School of Medicine, Washington, DC USA; 2 Department of Pharmacy and Therapeutics, Center for Clinical Pharmaceutical Sciences, School of Pharmacy, University of Pittsburgh, Pittsburgh, PA USA; 3 Department of Medicine, Renal-Electrolyte Division, School of Medicine, University of Pittsburgh, Pittsburgh, PA USA; 4 Department of Medicine Georgetown University School of Medicine, Washington, DC USA; 5 Center for the Study of Biological Complexity, Virginia Commonwealth University, Richmond, VA USA; 6 Department of Microbiology and Immunology, Medical College of Virginia Campus of Virginia Commonwealth University, Richmond, VA USA; 7 Departments of Pathology and Medicine, The George Washington University, Washington, DC USA; 8 Medical Faculty Associates, Department of Surgery, George Washington University School of Medicine & Health Sciences, Washington, DC USA

## Abstract

A mechanistic link between trimethylamine N-oxide (TMAO) and atherogenesis has been reported. TMAO is generated enzymatically in the liver by the oxidation of trimethylamine (TMA), which is produced from dietary choline, carnitine and betaine by gut bacteria. It is known that certain members of methanogenic archaea (MA) could use methylated amines such as trimethylamine as growth substrates in culture. Therefore, we investigated the efficacy of gut colonization with MA on lowering plasma TMAO concentrations. Initially, we screened for the colonization potential and TMAO lowering efficacy of five MA species in C57BL/6 mice fed with high choline/TMA supplemented diet, and found out that all five species could colonize and lover plasma TMAO levels, although with different efficacies. The top performing MA, *Methanobrevibacter smithii*, *Methanosarcina mazei*, and *Methanomicrococcus blatticola*, were transplanted into Apoe^−/−^ mice fed with high choline/TMA supplemented diet. Similar to C57BL/6 mice, following initial provision of the MA, there was progressive attrition of MA within fecal microbial communities post-transplantation during the initial 3 weeks of the study. In general, plasma TMAO concentrations decreased significantly in proportion to the level of MA colonization. In a subsequent experiment, use of antibiotics and repeated transplantation of Apoe^−/−^ mice with *M*. *smithii*, led to high engraftment levels during the 9 weeks of the study, resulting in a sustained and significantly lower average plasma TMAO concentrations (18.2 ± 19.6 μM) compared to that in mock-transplanted control mice (120.8 ± 13.0 μM, p < 0.001). Compared to control *Apoe*^*−/−*^ mice, *M*. *smithii*-colonized mice also had a 44% decrease in aortic plaque area (8,570 μm [95% CI 19587–151821] vs. 15,369 μm [95% CI [70058–237321], p = 0.34), and 52% reduction in the fat content in the atherosclerotic plaques (14,283 μm [95% CI 4,957–23,608] vs. 29,870 μm [95% CI 18,074–41,666], p = 0.10), although these differences did not reach significance. Gut colonization with *M*. *smithii* leads to a significant reduction in plasma TMAO levels, with a tendency for attenuation of atherosclerosis burden in Apoe^−/−^ mice. The anti-atherogenic potential of MA should be further tested in adequately powered experiments.

## Introduction

Atherosclerotic vascular disease is the leading cause of death in the US^1^. The gut microbiome is now recognized as a mediator of numerous host physiological processes. Changes in the gut microbiome have been causally linked to several metabolic, inflammatory, and cardiovascular diseases, including atherosclerosis^2,3^. Systemic concentrations of the gut microbe-derived metabolite, trimethylamine-N-oxide (TMAO), is associated with atherosclerosis and major adverse cardiovascular events^4–6^. Choline diet dependent enhancement in atherosclerosis could be prevented by either gut microbiota suppression with broad spectrum antibiotics^6^, or in more recent studies, administration of an inhibitor of microbial choline trimethylamine (TMA) lyase activity and therefore TMAO generation (4,4-dimethyl-1-butanol)^7^, indicating a causal link between TMAO and atherosclerosis. In this study, we tested a novel microbiota-based approach to reduce systemic exposure of TMAO.

Gut microbes generate TMA from ingested precursors, including choline^6^, carnitine^4^, phosphatidylcholine^5^, betaine^8^, and trimethyllysine^9^. TMA is converted to TMAO by the host hepatic flavin monooxygenase 3 (FMO3)^10^. Multiple phylogenetically distinct bacteria are involved in TMA production^11^. There are many microbial families, genus, and species which possess microbial enzymes that can make TMA. There have been three microbial enzyme genes/sources of TMA thus far identified; the cutC/D^11^, the cntA/B gene^12^, and the YeaW/Z gene^13^.

In humans, FMO3 gene mutations cause the inherited disorder primary trimethylaminuria (TMAU)^14^, also known as the fish odor syndrome, which severely reduces the ability to convert TMA to TMAO. Consequently, the affected individuals excrete large amounts of odorous TMA in their urine, sweat, and breath^15^. Although the disorder is not known to affect patient health, it can have profound social and psychological consequences. Development of a probiotic that can catalytically consume TMA could also serve as a therapeutic approach for treating this genetic disorder.

Methanogenic archaea (MA) represents a distinct group of anaerobic archaea that produce methane as the end-product of their anaerobic respiration. The abundance and diversity of gut MA in humans is highly variable and limited to a few species^16–18^. *Methanobrevibacter smithii* is the dominant methanogen in the human gut, detected in 95.7% of individuals, whereas *Methaomassiliicoccus luminyensis* is detected only in 4% of individuals^19^. Certain MA such as *Methanosarcina* species are known to use methylated amines as growth substrates^20,21^. A catabolic microbe that literally consumes TMA preventing/intercepting TMA prior to when it can be absorbed and converted into TMAO, would serve as a therapeutic intervention. However, up until now this potential has largely remained a theoretical concept. However, up until now this potential has largely remained a theoretical concept. In this study, we tested the effect of MA colonization on blood TMAO level and atherosclerosis burden in the atherosclerosis prone *Apoe*^*−/−*^ mouse model.

## Materials and Methods

All methods were performed in accordance to guidelines and regulations, and under approval of the Institutional Biosafety Committee of The George Washington University.

### Methanogenic archaea (MA)

Selected species of known human gut and non-gut MA including: (a) *Methanobrevibacter smithii*, strain PS (DSM-86), (b) *Methaomassiliicoccus luminyensis*, strain B10 (DSM-25720), (c) *Methanosarcina mazei*, strain S-6 (DSM-2053), (d) *Methanoimicrococcus blatticola*, strain PA (DSM-13328) and (e) *Methanohalophilus portucalensis*, strain FDF-1 (DSM-7471) were obtained from DSMZ (Germany). *M*. *smithii and M*. *luminyensis* were selected because these are indigenous human gut MA^22^. For comparison, we selected *M*. *blatticola* (isolated from the hindgut of cockroach *Periplaneta americana*)^23^, *M*. *mazei* (isolated from a laboratory digester)^24^, and *M*. *portucalensis* (isolated from sediments at a saline lake)^25^.

### Animal Studies

All animal studies were performed under approval of the Animal Research Committee of The George Washington University. To test the efficacy of MA to colonize gut and metabolize TMA, 4-week-old female C57BL6J (Jackson Laboratory, Bar Harbor, Maine) were randomly selected for transplantation with one of the MA or sham, and placed on high TMA/choline diet. Because of the small sample size (n = 3/group), considering that the male mice have lower plasma TMAO levels, we used only female mice in this pilot study^6,10^. Prior to transplantations, endogenous gut microbiota were suppressed using an oral cocktail of poorly absorbed antibiotics previously reported to suppress TMA and TMAO levels^6^ (0.5 g/L vancomycin, 1 g/L neomycin sulfate, 1 g/L metronidazole, 1 g/L ampicillin) administered in drinking water ad lib for three weeks, refreshed 3 times per week. Each mouse was inoculated with a single gavage of 10^8^ MA 24 hours after the antibiotic treatment was completed. The test groups (n = 3/group) received a 0.1 ml gavage of corresponding MA in liquid growth media. The control groups (n = 3 each) received a 0.1 ml gavage of liquid growth media. Following transplantations, mice were supplemented with choline (1.0%, Sigma Aldrich) and TMA (0.12%, Sigma Aldrich) in drinking water ad lib, refreshed 3 times per week. The negative control group (NC) received untreated water. Both choline and TMA were given to animals since antibiotic treatment depletes most of the TMA-generating bacteria. All animals received standard chow diet for the entire study. Blood plasma and fresh stool samples were collected from the mice on days 2, 10, and 30 post-transplantation and stored at −80 °C, until processed. In a second study, 4-week-old female C57BL6J mice with Apoe^−/−^ background (n = 5/group) (Jackson Laboratory) were treated with antibiotics, transplanted with *M*. *smithii*, *M*. *mazei*, and *M*. *blatticola* and fed with high TMA/choline diet as described above to determine the impact of MA on TAMO concentration. In a third study, we examined the effect of sustained high colonization with *M*. *smithii* on TMAO in *Apoe*^*−/−*^ mice (n = 5/group). The experiment was performed as described above, except that *M*. *smithii* transplantation was repeated every three weeks, until the end of the study. One group of mice (n = 5) was maintained throughout the study on an antibiotic regimen (0.5 g/L vancomycin and 1 g/L ampicillin) to which MA are resistant, in order to chronically suppress the endogenous gut microbiota and enhance MA colonization (Table 1).

**Table 1:** Animal experiments study design.

Mouse experiment	Mouse model	Archaea species studied	^#^of mice/group (N)	Duration of study (weeks)	Transplant frequency	Antibiotic treatment
1	C57BL/6	MS, MM, MB, ML, MP	3	4	Once	Pre-transplant
2	*Apoe* ^*−/−*^	MS, MM, MB	5	4	Once	Pre-transplant
3	*Apoe* ^*−/−*^	MS	5	12	Every 3 weeks	Pre- and post-transplant

MS, *M. smithii*; MM, *M. mazei*; MB, *M. blatticola*; ML, *M. luminyensis*; MP, *M. portucalensis*.

### Quantification of MA

Gut colonization by the MA was determined using quantitative real-time PCR (q-PCR). Briefly, microbial DNA was extracted from stool samples using the Qiagen DNeasy PowerSoil kit. The stool specimens were tested for the levels of engraftment by q-PCR as previously described^26^. Arch344f (ACGGGGYGCAGCAGGCGCGA)^26^ and Arch806r (GGACTACCCGGGTATCTAAT)^27^ primers were used to target the archaea 16 S rRNA gene present in MA. Q-PCR was performed for detection and quantification of microbes using SYBR Green PCR Master Mix (Applied Biosystems, Foster City, CA) in an Applied Biosystems 7900HT Sequence Detection system (Applied Biosystems)^28^. PCR products amplified from the bacterial and archaeal 16 s rRNA genes were cloned and grown in transformed *E*. *coli* to establish a standard curve, as described previously^29^. The accuracy of the q-PCR assay was confirmed through melting curve analysis and agarose gel electrophoresis. Calibration curves were obtained using 10-fold serial dilutions of known concentrations of cloned DNA samples that were prepared in the laboratory.

### 16S rRNA-based taxonomic survey

The V4 region of the 16 S rRNA gene was PCR amplified using barcoded primers^30^ essentially as previously described^31^. Briefly, for each reaction, 2 μL of extracted DNA was combined with 12.5 μL 2X Phusion Hot Start II High-Fidelity PCR Master Mix (Thermo Fisher Scientific), and 100 nM each of forward and reverse primers. The primers were designed containing the Illumina linker adaptor, a unique index sequence, followed immediately by a variable sequence spacer (0–6 bases) and 16 S rRNA gene primers (Table 2). The PCR was carried out in a 25 µl reaction in a Thermal Cycler (Applied BioSystem GeneAmp PCR system 9700) with the following parameters: initial denaturation at 94 °C for 3 min., followed by 35 cycles of 94 °C for 45 s, 50 °C for 60 s, and 72 °C for 90 s with a final extension at 72 °C for 10 min. The PCR was performed in triplicates with two PCR controls, a negative water control and a positive Mock Community control (M.G. Serrano, unpublished). All amplicons were quantified using Picogreen (Invitrogen/Thermo Scientific) and a spectrofluorimeter (Biotek). Amplicons were combined in equal volumes, followed by removal of unincorporated primers, salts and enzymes using Agencourt AMpure XP beads as described by the manufacturer. The DNA concentration of this concentrated pool was confirmed by qPCR using the KAPA Library Quantification Kit for Illumina platform. The library pool was diluted and denatured as described in the Illumina MiSeq library preparation guide. The sequencing run was conducted on the Illumina MiSeq using 600 cycles reagent kit (version 3) and 2 × 300 b paired end sequencing. Demultiplexing of sequence reads was performed using an in-house Python script. Raw paired-end sequence data was merged and quality-filtering using the MeFiT software^32^ which invokes CASPER^33^ for merging paired-end sequences and quality filters them using a *meep-score* (maximum expected error rate) cutoff of 1.0. Non-overlapping high quality paired end reads were retained for downstream analysis by linking them artificially with 15 *N*s. High-quality sequences were taxonomically assigned using the Ribosomal Database Project (RDP) Naïve Bayesian Classifier (version 2.9)^34^ with a bootstrap cutoff of 80%. PICRUSt (Phylogenetic Investigation of Communities by Reconstruction of Unobserved States)^35^ was used to infer the abundance of metabolic pathways from 16 S rRNA gene sequencing data. The predicted functions (KOs) were collapsed into hierarchical KEGG (Kyoto Encyclopedia of Genes and Genomes) pathways using the categorize_by_function step provided in the PICRUSt pipeline.

**Table 2:** PCR primers for amplification of V4 region of 16 S rRNA gene.

v4L_BT517	AATGATACGGCGACCACCGAGATCTACACTCTTTCCCTACACGACGCTCTTCCGATCTACGACGTGGTGYCAGCMGCCGCGGTAA
v4L_13_R	CAAGCAGAAGACGGCATACGAGATGGACTTCCAGCTGTGACTGGAGTTCAGACGTGTGCTCTTCCGATCTCCGYCAATTYMTTTRAGTTT
v4L_14_R	CAAGCAGAAGACGGCATACGAGATCTCACAACCGTGGTGACTGGAGTTCAGACGTGTGCTCTTCCGATCTCCGYCAATTYMTTTRAGTTT
v4L_15_R	CAAGCAGAAGACGGCATACGAGATCTGCTATTCCTCGTGACTGGAGTTCAGACGTGTGCTCTTCCGATCTCCGYCAATTYMTTTRAGTTT
v4L_16_R	CAAGCAGAAGACGGCATACGAGATATGTCACCGCTGGTGACTGGAGTTCAGACGTGTGCTCTTCCGATCTCCGYCAATTYMTTTRAGTTT
v4L_17_R	CAAGCAGAAGACGGCATACGAGATTGTAACGCCGATGTGACTGGAGTTCAGACGTGTGCTCTTCCGATCTCCGYCAATTYMTTTRAGTTT
v4L_18_R	CAAGCAGAAGACGGCATACGAGATAGCAGAACATCTGTGACTGGAGTTCAGACGTGTGCTCTTCCGATCTCCGYCAATTYMTTTRAGTTT
v4L_19_R	CAAGCAGAAGACGGCATACGAGATTGGAGTAGGTGGGTGACTGGAGTTCAGACGTGTGCTCTTCCGATCTCCGYCAATTYMTTTRAGTTT
v4L_20_R	CAAGCAGAAGACGGCATACGAGATTTGGCTCTATTCGTGACTGGAGTTCAGACGTGTGCTCTTCCGATCTCCGYCAATTYMTTTRAGTTT
v4L_21_R	CAAGCAGAAGACGGCATACGAGATGATCCCACGTACGTGACTGGAGTTCAGACGTGTGCTCTTCCGATCTCCGYCAATTYMTTTRAGTTT
v4L_22_R	CAAGCAGAAGACGGCATACGAGATTACCGCTTCTTCGTGACTGGAGTTCAGACGTGTGCTCTTCCGATCTCCGYCAATTYMTTTRAGTTT
v4L_23_R	CAAGCAGAAGACGGCATACGAGATTGTGCGATAACAGTGACTGGAGTTCAGACGTGTGCTCTTCCGATCTCCGYCAATTYMTTTRAGTTT
v4L_24_R	CAAGCAGAAGACGGCATACGAGATGATTATCGACGAGTGACTGGAGTTCAGACGTGTGCTCTTCCGATCTCCGYCAATTYMTTTRAGTTT

### Plasma TMAO measurement

Ultra performance liquid chromatography-tandem mass spectrometry (UPLC-MS/MS) was used to measure plasma TMAO concentrations as described previously with minor modification^36^. Briefly, the UHPLC-MS/MS system consisted of an Acquity UPLC I-class sample mannager (Waters, Milford, MA), Acquity UPLC I-class binary solvent mannager, and a TSQ Quantum Ultra triple quadrupole mass spectrometer (Thermo, San Jose, CA). For protein precipitation, plasma (50 µL) was combined with an internal standard (200 µL of *d9*-TMAO 0.5 µg/mL) in methanol. After centrifugation, 20 µL of supernatant was diluted with 100 µL of 75:25 acetonitrile:methanol and 5.0 µL of this mixture was injected onto the UPLC-MS/MS system. The standard curve ranged from 0.010–5.00 µg/mL (0.13–66.6 µM). The within-run and between-run precision (percent coefficient of variation) was <15%.

### Assessment of atherosclerosis

At the end of each study, mice were euthanized, and the heart was removed just proximal to the aortic arch. The heart was cut transversely at the level of the atria and placed ventricle down into a tissue mold in optimal cutting temperature compound (OCT compound) and stored at −20 °C until sectioning. Multiple cryosections at 10 μ thickness were taken of the aortic sinus and aortic root and stained for fat content with Oil-Red-O (ORO). Six sections from each heart were selected for image analysis, including the first section that contained the three leaflets of the aortic valve and the next 5 sections at 40 μ intervals along the aortic root distal to the valve over a total distance of 200 μ. The area of the plaque and the content of ORO was quantified by image analysis (ImageJ software) and calculated as an average of the area of plaque found in each of the 6 individual aortic valve sections taken for analysis^37^. Two animals from the negative control group and one of the transplanted animals died prior to obtaining samples for atherosclerosis evaluation.

### Statistical Analyses

We used analysis of variance and Student’s t test to compare mean plaque size between groups. A random effects mixed model was used to examine the species main effect (with levels *M*. *smithii*, *M*. *mazei*, and *M*. *blatticola*, *M*. *luminyensis*, *M*. *portucalensis*, and control), the time main effect (day 2, 10, 30), and the species x time interaction Values are presented as mean ± SD, and those with a value of P < 0.05 were considered significant. Data analysis was performed using SAS 9.4.

## Results

### MA can colonize normal C57BL/6 mice guts and lower their plasma TMAO concentrations

**Figure 1:**
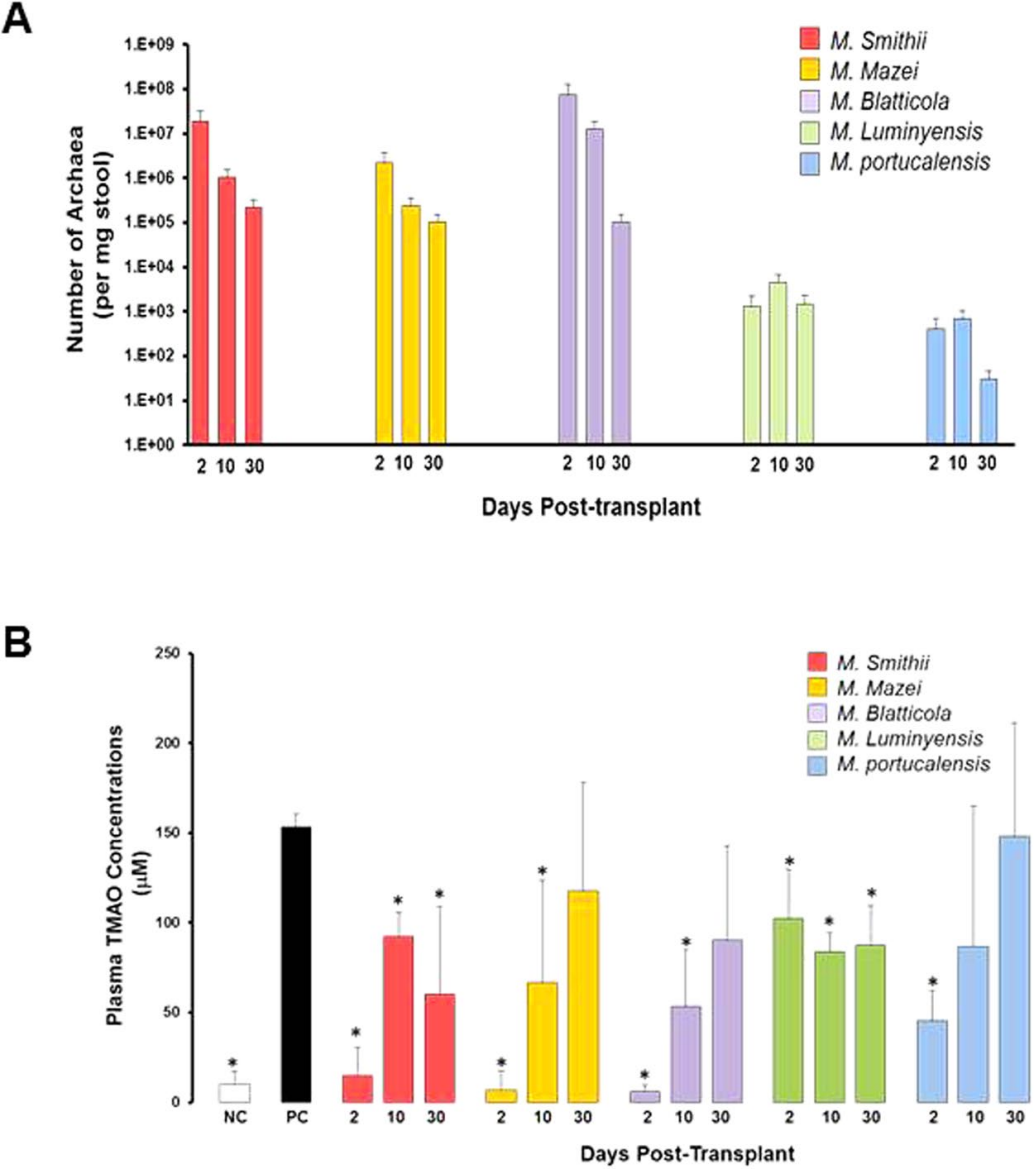
MA can colonize normal C57BL/6 mice guts and lower their plasma TMAO concentrations. (**A**) Q-PCR analysis of fecal samples from C57BL/6 mice transplanted with one of five different representative gut- and non-gut MA, namely: *M*. *smithii*, *M*. *mazei*, *M*. *blatticola*, *M*. *luminyensis*, and *M*. *portucalensis*. Stool samples were collected at days 2, 10, and 30 post-transplantation. (**B**) Plasma TMAO concentrations in mock- and MA-transplanted C57BL/6 J mice collected at days 2, 10 and 30 post-transplantation. Mock-transplanted negative control mice (NC) only received regular water, whereas mock-transplanted positive control mice (PC) received high choline/TMA water, similar to MA-transplanted mice.

In an initial experiment, we screened five MA for colonization efficacy and TMAO lowering potential using C57BL/6 mice. *M*. *smithii*, *M*. *mazei*, and *M*. *blatticola* had the highest engraftment levels ranging from 1.9 × 10^7^ to 7.4 × 10^7^ copies/mg stool on day 2 post-transplant, which gradually decreased (1.0 × 10^5^ to 2.2 × 10^5^ copies/mg stool) on day 30 post-transplant (Fig. 1A). *M*. *luminyensis* and *M*. *portucalensis* had the lowest engraftment levels ranging from 0.4 × 10^3^ to 1.3 × 10^3^ copies/mg stool on day 2 post-transplant, which also decreased on the 30^th^ day.

The plasma TMAO concentration was markedly elevated in the group of mice that were on the choline/TMA-supplemented water (positive control) compared to the negative control mice fed with regular water (153.6 ± 2.8 vs. 10.1 ± 7.0 μM, p < 0.001). On day 2 post-transplant, plasma TMAO concentrations were significantly lower in mice colonized with *M*. *smithii* (14.8 ± 15.7 μM, p = 0.003), *M*.*mazei* (6.9 ± 10.6 μM, p < 0.001), *M*. *blatticola* (5.9 ± 3.8 μM, p < 0.001), *M*. *luminyensis* (102.3 ± 27.3 μM, p = 0.03), and *M*. *portucalensis* (45.57 ± 16.8 μM, p < 0.01), compared to the positive control mice (Fig. 1B**)**. TMAO concentrations showed a rebound increase from day 10 post-transplantation, which paralleled the decline in MA colonization levels.

### MA can colonize Apoe^*−/−*^ mice guts and lower their plasma TMAO concentrations

**Figure 2:**
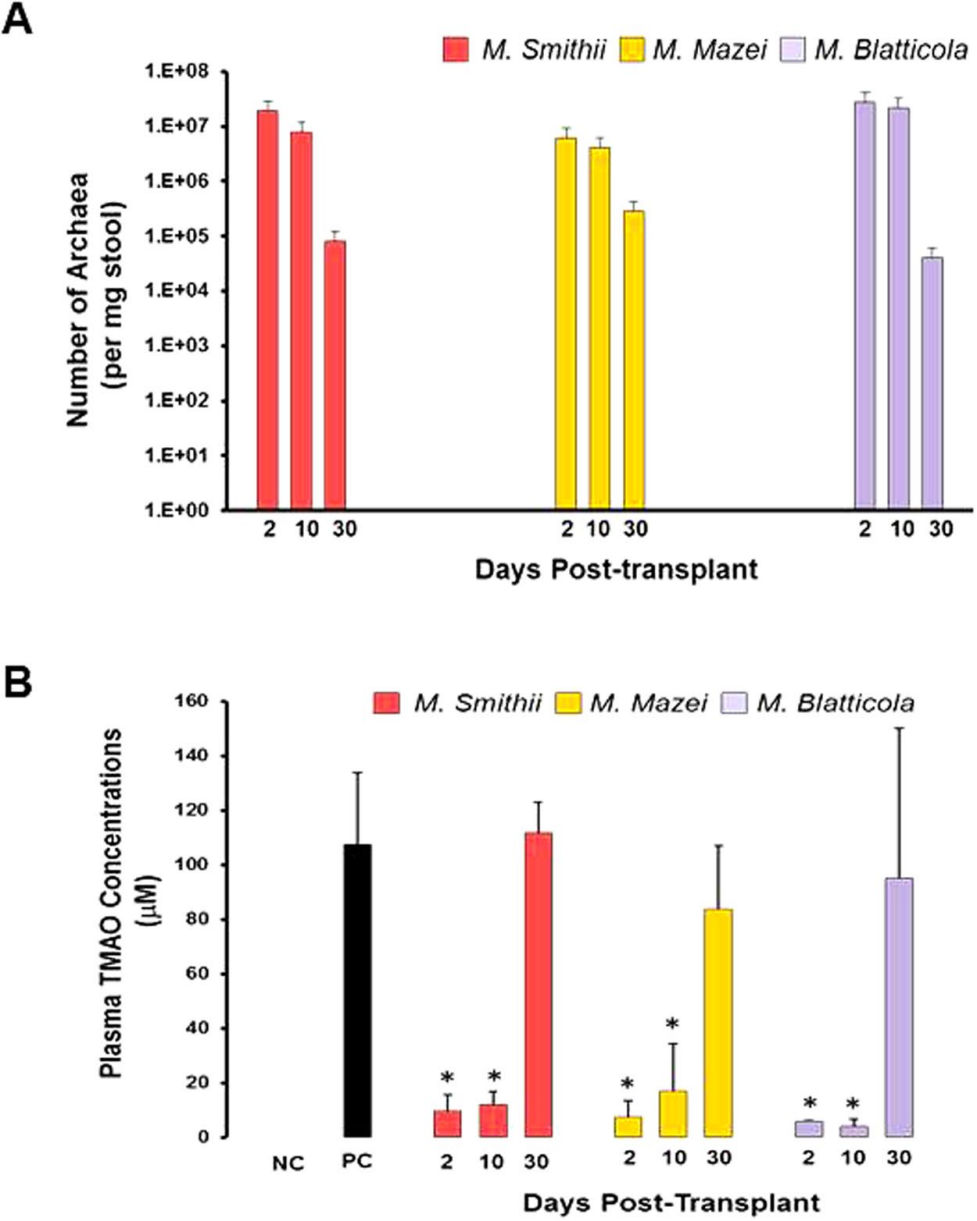
MA can colonize Apoe^*−/−*^ mice guts and lower their plasma TMAO concentrations. (**A**) Q-PCR analysis of fecal samples from *Apoe*^*−/−*^ mice transplanted with one of the 3 different MA, namely: *M*. *smithii*, *M*. *mazei*, *and M*. *blatticola*. Stool samples were collected at days 2, 10, and 30 post-transplantation. (**B**) Plasma TMAO concentrations in mock- and MA-transplanted *Apoe*^*−/−*^ mice collected at days 2, 10 and 30 post-transplantation. Mock-transplanted negative control mice (NC) only received regular water, whereas mock-transplanted positive control mice (PC) received high choline/TMA water, similar to MA-transplanted mice.

Based on their performance in C57BL/6 mice, three of the best performing MA, *M*. *smithii*, *M*. *mazei*, and *M*. *blatticola*, were selected and tested in *Apoe*^*−/−*^ mice. In general, engraftment of the 3 transplanted MA in *Apoe*^*−/−*^ mice were similar to that obtained in C57BL/6 mice, ranging from 0.6 0 × 10^7^ to 3.0 × 10^7^ copies/mg stool on day 2 post-transplant (Fig. 2A). TMAO concentrations in mice colonized with *M*. *smithii (*9.5 ± 5.9 μM), *M*. *mazei* (7.4 ± 6.1 μM) and *M*. *blatticola* (5.8 ± 0.6 μM) were significantly lower on day 2 compared to positive control mice (98.5 ± 26.5 μM) (Fig. 2B). The plasma TMAO concentrations remained low up to 10 days post-transplantation. The group x time interaction was significant (p = 0.004), indicating that the pattern of TMAO across time differed by species.

### Repeated transplantation of Apoe^*−/−*^ mice with *M*. *smithii* in presence of antibiotics leads to stable and high level gut colonization and diminishes the plasma TMAO concentrations

**Figure 3:**
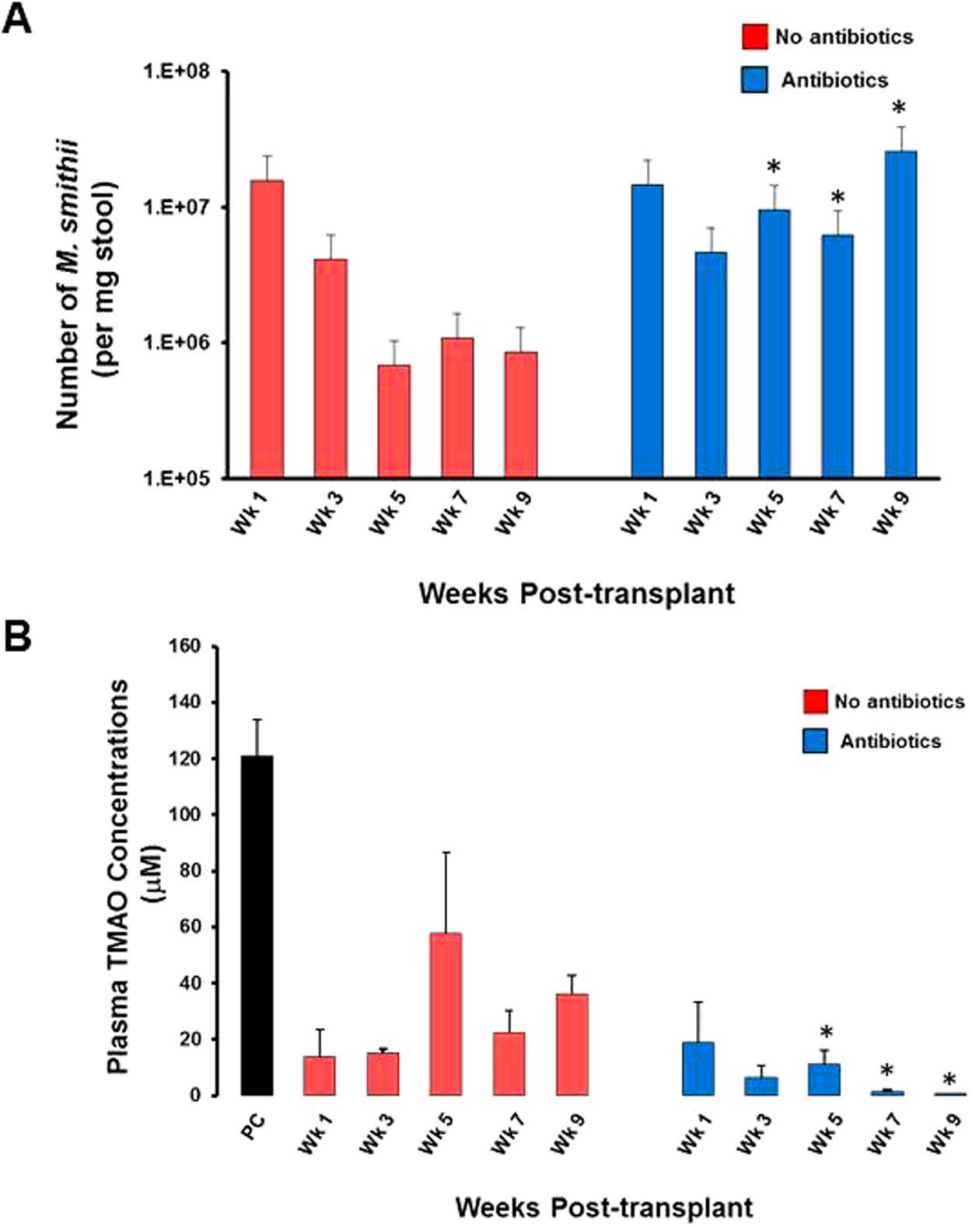
Repeated transplantation of Apoe^*−/−*^ mice with *M*. *smithii* in presence of antibiotics leads to stable and high level gut colonization and diminishes the plasma TMAO concentrations. (**A**) To maintain a high level of gut colonization with the MA, *M*. *smithii*, transplantation was repeated every 3 weeks for the duration of the study. A second group of mice (+Antibiotics) was also maintained on vancomycin and ampicillin, in addition to receiving repeated transplantations. Weekly blood and stool samples were collected from the mice for 9 weeks. Stool samples were analyzed by q-PCR for MA engraftment levels. (**B**) Plasma TMAO concentrations in mock- and *M*. *smithii*-transplanted *Apoe*^*−/−*^ mice (±antibiotics) collected weekly for 9 weeks post-transplantation. Mock-transplanted positive control mice (PC) received high choline/TMA water, similar to *M*. *smithii*-transplanted mice, but not antibiotics.

In order to improve gut colonization efficacies, we tested whether repeated transplantation with *M*. *smithii* leads to sustained high colonization level and persistent reduction in TMA concentration in *Apoe*^*−/−*^ mice. In one group of repeatedly transplanted mice (n = 5/group) we also examined whether chronic suppressive antibiotic therapy further enhances colonization with *M*. *smithii*. As shown in Fig. 3A, repeated transplantation of *M*. *smithii* led to high engraftment levels throughout the study period. High and stable gut colonization with *M*. *smithii* led to lower average plasma TMAO concentrations (18.2 ± 19.6 μM) throughout the study period compared to that in positive control mice (120.8 ± 13.0 μM, p < 0.001) (Fig. 3B). The group of mice which were maintained on antibiotics, in addition to repeated transplantations, had a higher MA engraftment level compared to the mice not receiving antibiotics (1.3 ± 7.7 × 10^7^ vs. 4.5 ± 6.4 × 10^6^ average copies/mg stool) and lower average TMA concentrations (7.41 ± 9.38 vs. 29.1 ± 31.2 μM, p < 0.001).

### Antibiotic depletion of Firmicutes enhances gut colonization by *M*. *smithii*

**Figure 4:**
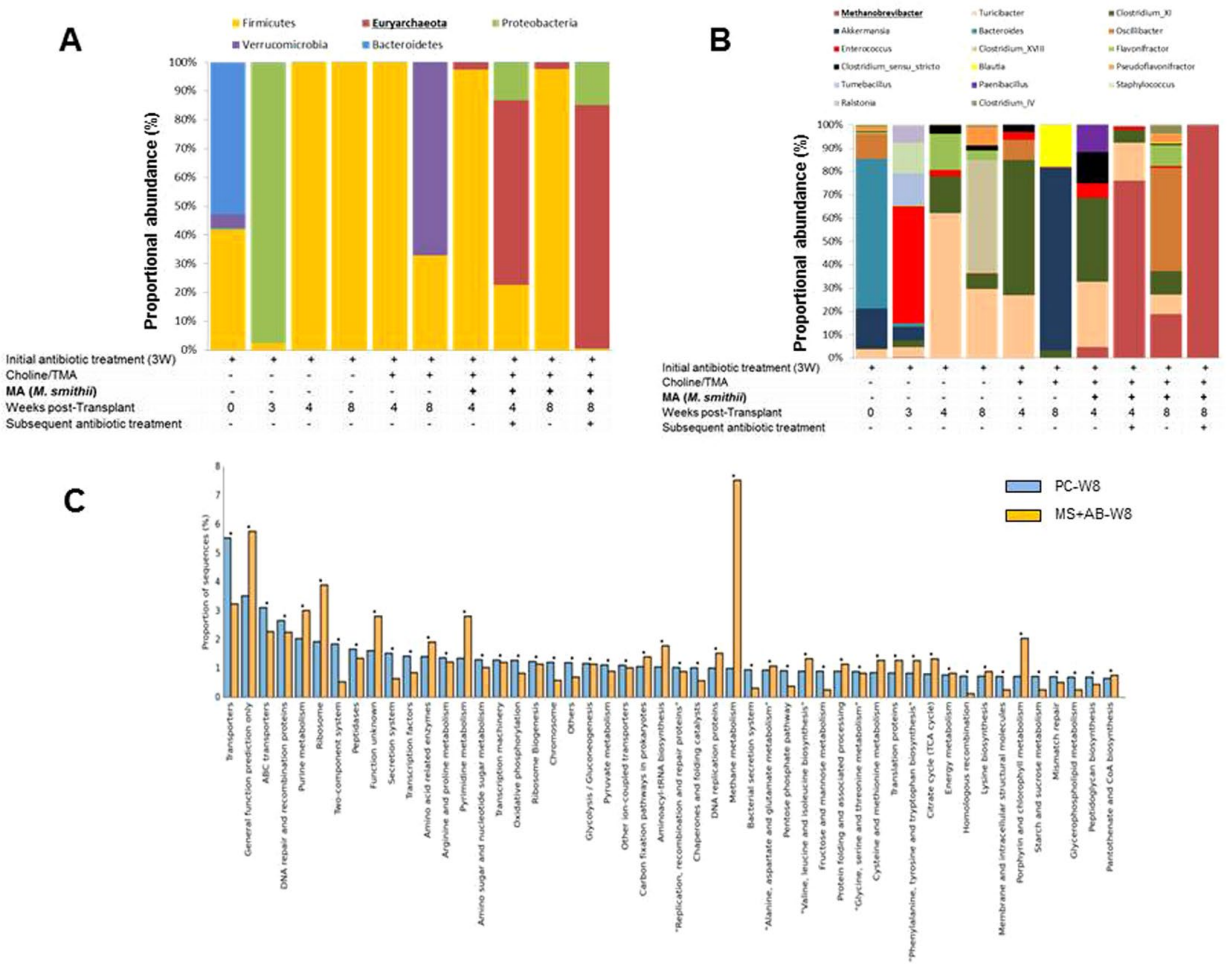
Antibiotic depletion of Firmicutes enhances gut colonization by *M*. *smithii*. *Apoe*^*−/−*^ mouse gut microbiome characterization based on 16 S rRNA gene sequencing. Shown is the histogram of proportional changes in gut microbiota OTU abundance at the (**A**) phylum and (**B**) genus levels as measured for the different groups (NC, PC, No-antibiotics, antibiotic). (**C**) Metabolic potential of *M*. *smithii*-colonized mice compared to un-transplanted mice.

16 S gut microbiome analysis of the stool samples from mice in the last experiment demonstrated that the baseline and pre-antibiotic microbiome profile of the mice consist of 53% Bacterioidetes, 43% Firmicutes, and 4.9% Verrucomicrobia (Fig. 4A). However, following the 3-week antibiotic treatment, the Bacterioidetes, Firmicutes and Verrucomicrobia levels decreased to 2.4%, 0.2%, and 0.1%, respectively, and Proteobacteria constituted the majority of the remaining bacteria that colonized in the gut (97.1%). In the negative control group, after stopping antibiotics, within 4 weeks, the Firmicutes returned and constituted the main surviving phylum (100%). Mice that were fed with Choline/TMA also showed 100% colonization by Firmicutes on week-4, but during the next 4 weeks, the level decreased to 32.8% with the return of Verrucomicorbia, which constituted 67.2% of the gut bacteria. Gut colonization levels with *M*. *smithii* were 2.4% and 2.2% in weeks 4 and 8, respectively in mice that were not on antibiotics, and 64% and 84% in weeks 4 and 8, respectively, in mice that received antibiotics.

At the genus level, the untreated mouse microbiome was dominated by *Bacteroides* at the baseline, while *Clostridium* and *Turicibacter* trended higher among the remaining bacteria in antibiotic-treated mice (Fig. 4B). Choline/TMA supplemented mouse microbiome was dominated by *Akkermansia*, which was replaced by *Clostridium* and *Turicibacter* in *M*. *smithii*-colonized mice. There was a significant difference in the colonization levels of *M*. *smithii* between the antibiotic treated and untreated mice, and the proportional distribution of classes of bacteria varied significantly. Functional analysis using PICRUSt^35^ showed that purine and pyrimidine metabolism, methane metabolism, and “phenylalanine, tyrosine and tryptophan biosynthesis pathways were active. (Fig. 4C).

### Stable *M*. *smithii* gut colonization trends towards attenuation of choline/TMA-enhanced atherosclerosis

**Figure 5:**
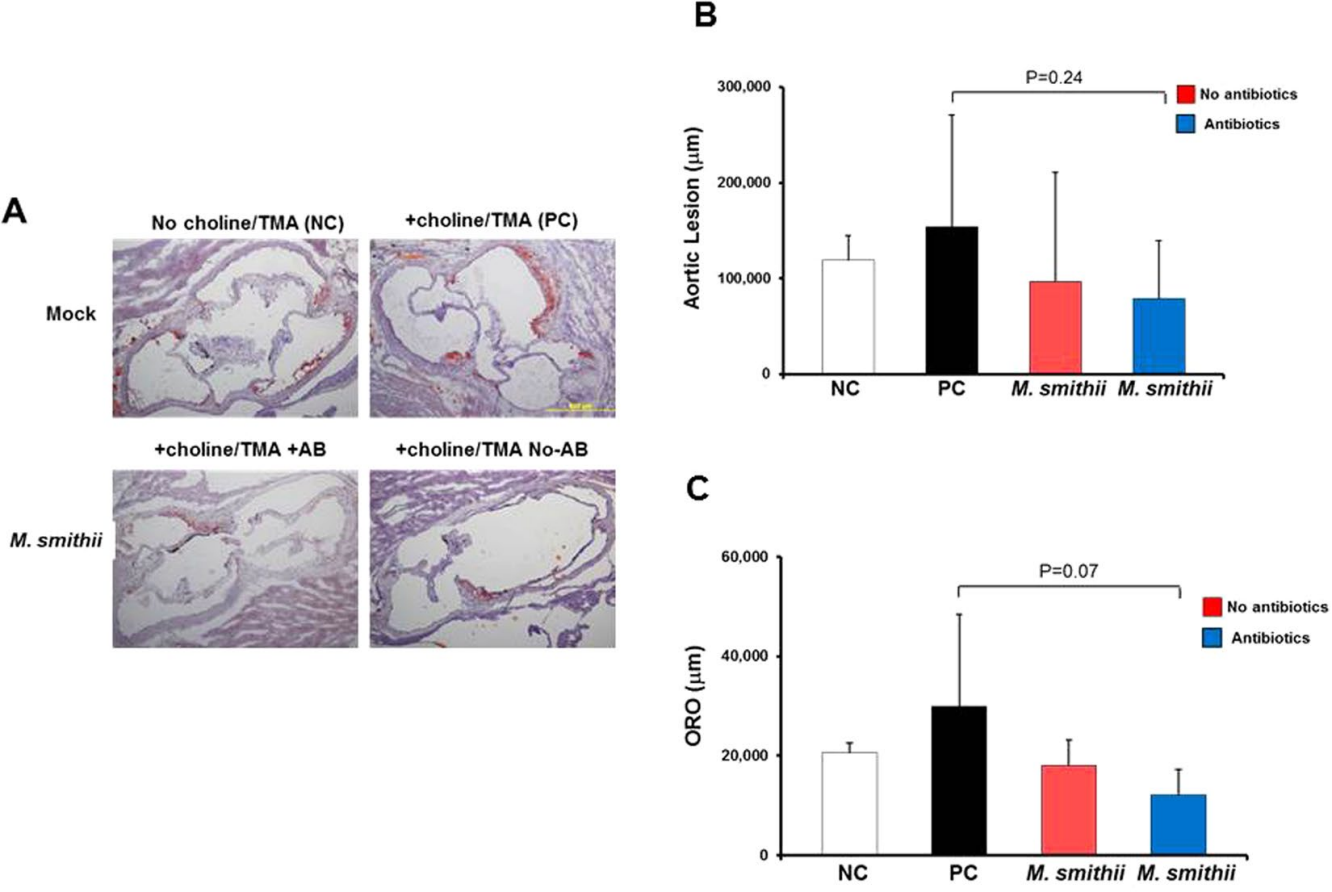
Stable *M*. *smithii* gut colonization trends towards attenuation of choline/TMA-enhanced atherosclerosis. (**A**) Representative Oil-Red-O (ORO)/hematoxylin staining of aortic root sections from 19-week-old female *Apoe*^*−/−*^ mice that were fed chemically defined chow (0.07% total choline), in the presence versus absence of choline/TMA (1.0% total choline; 1.2% TMA) provided in the drinking water, and either mock- or *M*. *smithii*-transplanted, as described in the Experimental Procedures. Scale bars, 500 μm. The AB-POS group was maintained on antibiotics in their drinking water for the duration of the experiment following transplantation. (**B**) Aortic root lesion plaque area and (**C**) ORO staining in the plaque area were quantified in 19-week-old female *Apoe*^*−/−*^ mice from the indicated diet and *M*. *smithii* transplanted groups. Data are presented as mean ± SE.

When examined for aortic root plaque size by histology, *M*. *smithii* colonized mice trended to lower plaque burden (85,704 μm [95% CI 19,587–151,821]) than the positive control mice (153,690 μm [95% CI [70,058–237,321]), but the differences did not reach statistical significance (p = 0.34) (Fig. 5). We also noted a 52.2% mean reduction in ORO staining area in *M*. *smithii* colonized mice (14,283 μm [95% CI 4957–23,608]) compared to that of the positive control mice (29,870 μm [95% CI 18,074–41,666]), which was also not statistically significant (p = 0.10).

## Discussion

In this study, we investigated the *in vivo* TMA-metabolizing efficacy of several gut and not-gut associated methyl-trophic methanoarchaea of the genera Methanomicrococcus (*Methanomicrococcus blatticola);* Methanosarcina (*Methanosarcina mazei*), Methanohalophilus (*Methanohalophilus portucalensis*), and Methanomassiliicoccus (*Methanomassiliicoccus luminyensis*). The ability of all of these species to grow on TMA in culture has been previously demonstrated^23,25,38,39^. Also included in this study, was *M*. *smithii*, a member of Methanobrevibacter genus which is the most predominant methanoarchaea in the human gut. Unlike the other methanoarchaea used in this study, *M*. *smithii* is not known to grow on TMA in culture. However, a recent study demonstrated that many essential genes involved in methanogenesis, including methyltransferases are present and significantly up-regulated *in vivo* when *M*. *smithii was* co-colonized with a prominent human gut symbiont^40^.

We show that gut colonization with MA, consistently and significantly reduces plasma TMAO concentrations in two different mouse models that were maintained on a high choline and TMA diet. The reduction in plasma TMAO concentrations, in general paralleled the abundance of MA in the gut. We initially screened five species of MA isolated from gut and non-gut environments and noted that the colonization efficacy and the TMAO lowering efficacy is highest with *M*. *smithii*, a normal inhabitant of the human gut. Unlike *M*. *smithii*, the other indigenous human gut methanoarchaea, *M*. *luminyensis*, was very poor at gut colonization in mice and lowering blood TMAO levels. This observation is however, in accordance with the reports on low prevalence of *M*. *luminyensis* which is detected only in 4% of individuals, compared to that of *M*. *smithii* which has a high prevalence of nearly 96%^19^.

16 s rRNA sequencing showed that antibiotics, choline/TMA and *M*. *smithii* colonization led to distinct change in microbiome profile and functional changes in gene expression. Compared to *Apoe*^*−/−*^ mice fed with high choline/TMA diet, mice colonized with *M*. *smithii* had 44.2% lower atherosclerotic burden and 52.2% reduced fat content in the atherosclerotic plaques, but these differences did not reach statistical significance possibly due to the small sample size.

Increased systemic exposure of TMAO is associated with atherosclerosis and major adverse cardiovascular events^4–6^. TMAO originates from the microbiota-dependent breakdown of dietary phosphatidylcholine to TMA. Functional studies have shown that TMAO decreases expression of bile acid transporters in the liver and reduces synthesis of bile acids from cholesterol^4^. TMAO also inhibits reverse cholesterol transport and promotes accumulation of cholesterol in macrophages through increasing cell surface expression of proatherogenic scavenger receptors CD36 and Scavenger Receptor A^6^, thus, creating foam cells that subsequently accumulate in the endothelial wall causing inflammation and plaque formation. Another mechanism related to atherosclerosis is the increase in thrombosis mediated by TMAO^41^.

It is therefore, not surprising that there is considerable interest in treatments designed to lower TMAO concentrations^7^. Targeting gut microbial production of TMA using a structural analog of choline, 3,3-dimethyl-1-butanol, inhibits TMA formation and lowers TMAO concentrations in mice fed a high-choline or L-carnitine diet^7^. 3,3-dimethyl-1-butanol inhibited choline diet-enhanced endogenous macrophage foam cell formation and atherosclerotic lesion development in *Apoe*^*−/−*^ mice^7^. In this study, we took a different approach and aimed at depleting TMA as it is being formed by MA. *In vitro* studies have shown that certain MA can utilize TMA as a substrate for growth^38^. However, this is the first *in vivo* study that examines the utility of MA in decreasing plasma TMAO concentrations.

We used a logical step-wise approach including screening, discovery and validation. For our initial screening, we chose normal C57/BL6 mice to evaluate the gut colonization capability and TMAO lowering efficacy of five selected species of MA. Subsequently, we tested the TMAO lowering efficacy of the three top performing MA in *Apoe*^*−/−*^ mice. In the final experiment, we examined the effect of repeated transplantation with *M*. *Smithii*, and chronic suppression of endogenous bacteria by antibiotics, on colonization efficacy, TMAO concentrations and atherosclerosis level in *Apoe*^*−/−*^ mice.

Our initial study in C57BL/6 mice showed that all MA tested were able to colonized the gut and reduce TMAO concentrations irrespective of their original habitat, but with large inter-individual variations. Efficient gut colonization by *M*. *smithii* was evident, but the colonization level of the other human gut MA, *M*. *luminyensis*, was poor. In the second experiment, a significant reduction in plasma TMAO concentrations were also observed in *Apoe*^*−/−*^ mice transplanted with *M*. *smithii* (90.4%), *M*. *mazei* (92.5%), and *M*. *blatticola* (94%) on the 2^nd^ day, compared to non-transplanted control animals fed with high choline/TMA diet. Our initial experiments in both C57BL/6 and *Apoe*^*−/−*^ mice revealed that, even after a 3-week-long antibiotic depletion of the gut bacteria, colonization was only transient with a single dose of MA. In a 3^rd^ experiment, we show that repeated transplantations significantly improved the gut colonization levels, which was further improved by antibiotic suppression of endogenous gut bacteria. There was a corresponding sustained reduction in TMAO concentrations. 16 S sequencing analysis confirmed that maintaining the transplanted mice on antibiotics greatly enhances *M*. *smithii* engraftment, but depleted Firmicutes and enriched Proteobacteria.

We chose to test *M*. *smithii* because it is known to be the most predominant MA in the human gut. *M*. *smithii thrives* in the distal intestine through its versatility in consuming the fermentation products made by saccharolytic bacteria, and by its ability to produce surface glycans and adhesion-like proteins^40^. *M*. *luminyensis* also colonizes human gut, although at much lower levels compared to *M*. *smithii*^19^. In human gut, TMA-metabolizing MA depend on the gut microbiota for converting choline to TMA which serves as a carbon-source for MA. In return, MA facilitates the continuation of bacterial fermentation process in the gut by metabolizing and removing H_2_.

On a chow diet, *Apoe*^*−/−*^ mice demonstrate a total cholesterol level >500 mg/dL, with fatty streaks first observed in the proximal aorta at 12 weeks of age, and fibrous plaques appearing at 20 weeks of age^42^. A Western diet^43^, or a choline-rich diet^6^, induce a marked increase in plaque size and aggressive plaque morphology. We found that gut colonization *with M*. *smithii* resulted in a tendency for reduced atherosclerosis burden in *Apoe*^*−/−*^ mice fed with high choline/TMA diet. The results were not statistically significant, possibly due to small number of animals studied. In addition, we cannot exclude the possibility that factors other than TMAO contribute to the pathogenesis of atherosclerosis, which were unmodified by this intervention^44^.

Since our data on the lowering of plaque characteristics were not statistically significant, either this study needs to be repeated in a much larger cohort of animals, or it could be concluded that lowering TMAO (as significant as our study demonstrates), does not lower TMAO-induced atherosclerosis in *Apoe*^*−/−*^ mice.

We acknowledge that this is a pilot study in small number of animals and that the study could have benefited from measurement of TMA and choline concentrations; however, the study has a number of strengths. Strengths include: (a) first study to explore the utility of gut colonization by MA to lower plasma TMAO concentrations; (b) the evaluation of several candidate gut and non-gut MA in two mouse models; (c) the examination of single vs. repeated transplantation effect of MA; and, (d) the determination of MA engraftment efficiency after suppression of the endogenous gut microbiome.

While we do not know and did not investigate the factors that support increased engraftment level of MA in mice maintained on antibiotics, we speculate that there may be other bacteria and/or bacteria-derived factors that would either compete with or inhibit MA gut colonization. Clearly, the use of antibiotic in this study was to enhance gut colonization in our mouse model and facilitate the study of MA impact on blood TMAO levels, and not a recommended method to enhance the MA gut colonization in human. Future studies are needed to identify the exact mechanism and factors that support or compete with MA engraftment in human gut, and the consequence of increased MA abundance on the human host physiology and health.

To date, the impact of methanogens on human physiology and health is poorly understood. Association between MA and inflammatory bowel disease, colorectal cancers and obesity has been reported, however, there is no convincing evidence supporting the pathogenic properties of the MA, beyond occasional reports of constipation^45,46^. *M*. *smithii* has been shown to enhance energy retrieval by another sacharolytic bacterial species when co-transplanted into germ-free mice^47^. A study of fecal DNA extracts from patients with colorectal cancer, polypectomised, irritable bowel syndrome and control subjects found no significant association between these diseases and the presence of MA^48^. There is also no conclusive evidence that MA is associated with colon cancer^49,50^.

To summarize, our study shows that gut colonization with certain MA reduces plasma concentrations of TMAO. Among the MA tested, *M*. *smithii*, an endogenous human gut MA, was most effective in lowering plasma TMAO levels in *Apoe*^*−/−*^ mice fed with high choline/TMA, and showed a tendency to attenuate atherosclerosis. The anti-atherogenic potential of the MA should be confirmed in studies using larger number of animals. The field of microbiome research is still nascent, but is evolving rapidly. Therapeutic use of specific toxin-degrading gut commensal microbes as probiotics to lower the levels of uremic toxins such as TMAO is a novel concept, but if proven effective, it will have a significant impact on cardiovascular disease and progression of chronic kidney disease. Safety of MA should be tested in pilot studies prior to launching efficacy studies in human subjects.
